# CO_2_ and O_2_ Separation Dual-Phase Membranes for Diesel Heavy-Duty Vehicles Applications

**DOI:** 10.3390/membranes15020049

**Published:** 2025-02-05

**Authors:** Eirini Zagoraiou, Luca Cappai, Anastasia Maria Moschovi, Gabriele Mulas, Iakovos Yakoumis

**Affiliations:** 1 Monolithos Catalysts & Recycling Ltd., 83 Vrilissou, 11476 Athens, Greece; zagoraiou@monolithos.gr (E.Z.); moschovi@monolithos.gr (A.M.M.); 2 Department of Chemical, Physical, Mathematical and Natural Sciences, University of Sassari, Via Vienna 2, 07100 Sassari, Italy; l.cappai@studenti.uniss.it (L.C.); mulas@uniss.it (G.M.)

**Keywords:** CO_2_ separation, O_2_ reduction, dual-phase membranes, carbonates, diesel engine, heavy-duty vehicles (HDVs), excess oxygen, Euro standards, automotive

## Abstract

Diesel-engine Heavy-Duty Vehicle (HDV) exhaust gas mixture contains pollutants including unburned hydrocarbons, carbon monoxide, nitrogen oxides, and particulate matter. A catalyst-based emission control system is commonly used to eliminate the above pollutants. However, the excess of oxygen that exists in the exhaust gasses of diesel engines hinders the efficient and selective reduction of nitrogen oxides over conventional catalytic converters. The AdBlue^®^ solution, which is currently used to eliminate nitrogen oxides, is based on ammonia. The latter is toxic in high concentrations. The aim of this work is to develop an Oxygen Reduction System (ORS) to remove oxygen from the exhaust gas of diesel engines, allowing the successful catalytic reduction of nitrogen oxides on a reduction catalyst without the need for ammonia. The ORS device consists of dense composite dual-phase membranes that allow the permeation of oxygen and carbon dioxide. Even though the oxygen concentration gradient across the membranes favors oxygen spontaneous diffusion from the atmosphere to the exhaust gas, the carbonate ion-based technology proposed herein utilizes the big difference in the concentration of carbon dioxide across the membrane to remove oxygen without any power consumption requirement. The results of this study are promising for the application of O_2_ reduction in diesel HDVs.

## 1. Introduction

Diesel engines exhibit high efficiency, durability, and reliability together with their low operating cost. These important features make them the most preferred engine, especially for Heavy-Duty Vehicles (HDVs) [1]. In addition to the widespread use of these engines with many advantages, they play an important role in environmental pollution problems worldwide. Diesel engines are considered as one of the largest contributors to environmental pollution caused by exhaust emissions, and they are responsible for several health problems as well. The major products of the complete combustion of petroleum-based fuels in an internal combustion engine are carbon monoxide (12%) and water (11%), oxygen (9%) with nitrogen from air comprising most (67%) of the remaining exhaust [2]. During the combustion, a low fraction of the nitrogen is converted to nitrogen oxides and some nitrated hydrocarbons. In addition, diesel engines are lean combustion engines. Lean-burn engine technology forces engine combustion to occur at very high air–fuel (A/F) ratios of 25:1 and higher, which produces oxygen-rich exhaust gasses [3].

The four main pollutant emissions from diesel engines are carbon monoxide (CO), hydrocarbons (HC), nitrogen oxides (NO_x_), and particulate matter (PM). Due to the adverse effects of diesel emissions on health and the environment, governments put forward requirements for permissible exhaust emission standards [1]. Europe has developed Euro standards which have continuously been making the emission limitations more stringent since 1993 with the Euro I to Euro VI that is in today [4].

To comply with the stricter regulation of exhaust emissions, the use of catalytic converters on the exhaust system of the vehicles was initiated. Catalytic converters are wash-coated ceramic monoliths with catalytic materials and have a honeycomb structure. The most common automotive catalytic converters for diesel engines are Diesel Oxidation Catalysts (DOCs), Selective Catalytic Reduction device (SCR), and Three-Way Catalysts (TWCs). Diesel Oxidation Catalysts (DOCs) are catalytic converters designed specifically for diesel engines and reduce CO, HC, and PM emissions, while SCRs are for the reduction of NO_x_ [5]. Three-Way Catalysts convert the three main pollutants (unburned HC, CO, and NO_x_) simultaneously [6]. Furthermore, in order for soot particles to be removed from the exhaust, the Diesel Particulate Filter (DPF) technology was introduced. The filter traps the particulate matter within a filter from the exhaust by passing the exhaust gasses through a ceramic wall flow filter [5]. Catalyzed DPFs have also been used for further soot oxidation or reduction of NO_x_ and oxidation [2].

Diesel vehicle exhaust gasses contain high oxygen (O_2_) concentrations of up to 10% [3]. Oxygen enrichment in diesel engines (within the combustion chamber) is one strategy to reduce exhaust gas emissions and optimize diesel engine combustion [7]. However, TWC and SCR devices suffer from serious problems such as low catalytic activities especially regarding NO_x_ reduction, narrow temperature windows, and insufficient durability when operating in excess oxygen.

Nitrogen oxides are harmful to human health and the environment. The removal of NO_x_ in an oxygen-rich exhaust is extremely difficult for the conventional TWC. This issue has prompted research on the development of a new catalyst technology that is capable of reducing NO_x_ in excess oxygen, that is, a NO_x_ storage–reduction (NSR) catalyst. However, these catalysts have several serious problems such as low catalytic activities, narrow temperature windows, and insufficient durability, among others [3].

Another process for controlling the emissions of nitrogen oxides is SCR’s technology which uses ammonia (NH_3_) as a reducing agent. Ammonia is introduced into the exhaust stream from a combustor. The control of the exhaust gas recirculation (EGR) and AdBlue amount allows the reduction in both NO_x_ emissions levels and the overall cost of diesel engines [8]. However, NH_3_ is a hazardous substance that requires cautious management. Instead of the direct utilization of ammonia, urea is used widely in a system known as the urea-SCR system. The urea hydrolysis and NO_x_ reduction reactions are shown by Equations (1)–(4) [9].

(1) $$CO({NH}_{2}{)}_{2}\;+H_{2}O\to 2{NH}_{3}+{CO}_{2}$$

(2) $$4NO+4{NH}_{3}+O_{2}\to 4N_{2}+6H_{2}O$$

(3) $$6NO+8{NH}_{3}^{-}\to 7N_{2}\;+12H_{2}O$$

(4) $$NO+{NO}_{2}+2{NH}_{3}^{-}\to 2N_{2}\;+3H_{2}O$$

The motivation for applying the technology is to meet and comply with the regulations, which have become progressively more stringent in each field [10].

The over-doping of urea, low temperatures in the system, and/or catalyst degradation may lead to NH_3_ emissions. Eventually, that concern led to the introduction of an ammonia emission limit for HDVs in the Euro VI standards ((EC) No 582/2011) [11,12].

**Figure 1:**
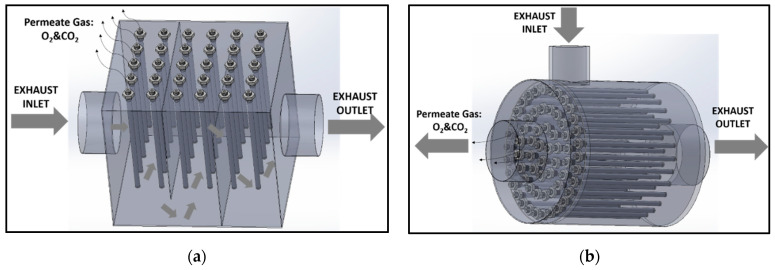
Oxygen Reduction System (ORS): (**a**) cubical layout of ORS; (**b**) cylindrical layout of ORS.

In this study, an Oxygen Reduction System (ORS) for the reduction of oxygen concentration in the lean exhaust mixture without the utilization of ammonia is proposed. The ORS would be in a multi-tubular membrane module formation as presented in Figure 1. The exhaust gas is fed through the external surface of these tubular hollow membranes and CO_2_ and O_2_ permeate across the membrane, while the total surface area should be adjusted to obtain an adequate permeation rate. The produced CO_2_ and O_2_ mixture can be either released into the atmosphere or used onboard to improve the efficiency of the engine utilizing the high O_2_ content [13].

**Figure 2:**
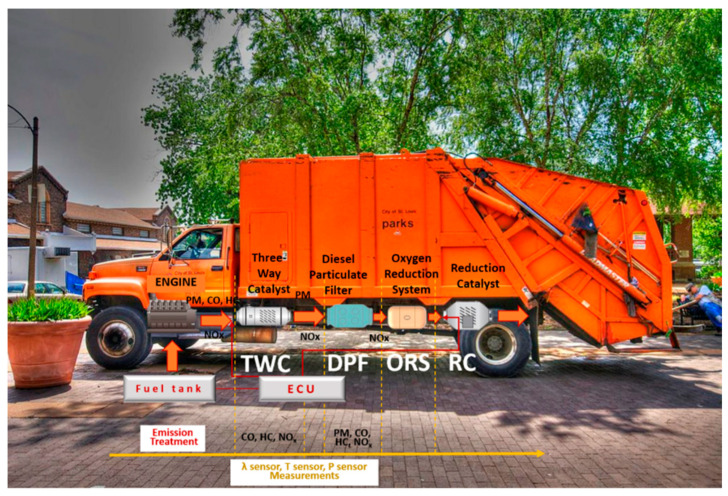
Catalyst-based emission control system (CECS) based on the technology proposed by this study.

This ORS will comprise part of a complete catalyst-based emission control system (CECS). CECS consists of a Three-Way Catalyst (TWC), a catalyzed Diesel Particulate Filter (c-DPF), an Oxygen Reduction System (ORS), and a reduction catalyst (RC) as shown in Figure 2. The catalysts (TWC, RC, and c-DPF) are synthesized with a patented method containing Copper (Cu), substituting the use of rare Platinum Group Metals (PGMs). More specifically, the TWC contains Cu/Pd/Rh, the reduction catalyst (RC) contains Cu/Rh, and the catalyzed DPF contains Cu/Pt [14]. The layout of these components will be placed in the exhaust system of HDVs as depicted in Figure 2 [15].

Dual-phase mixed electron-carbonate ion conducting membranes for perm-selective separation of CO_2_ attracted more and more interest from their first introduction [16]. Research studies regarding the optimization of the structure, geometry, and performance of the membrane; the importance of the surface and support material selection; and modifications of the physical and chemical properties of molten carbonate salt have been addressed to fabricate efficient and durable CO_2_ separation membranes [17,18,19]. Metal-carbonate membranes typically consist of a molten carbonate salts’ mixture phase supported on a porous electronically conducting metal substrate. Herein, membranes with an alkali or/and alkaline earth molten carbonate (MC) phase in a porous stainless steel (SS316) support were synthesized by a direct infiltration method [16].

The mechanism is in regard to a dual-phase metal-carbonate ion-based membrane consisting of two phases conducting electrons and ${CO}_{3}^{2-}$, respectively, for the selective permeation of both O_2_ and CO_2_. Carbonate ion-based membranes are permeable to O_2_ and CO_2_ (1:2 ratio) and the semi-reactions (Equations (5)–(7)) that take place on the two sides of the membrane are as follows:

(5) $${CO}_{2}+\frac{1}{2}O_{2}+2e^{-}\to {CO}_{3}^{2-}$$

(6) $${CO}_{3}^{2-}\to {CO}_{2}+\frac{1}{2}O_{2}+{2e}^{-}$$

(7) $$\mathrm{Total}:\;\frac{1}{2}O_{2}+{CO}_{2}+{2e}^{-}↔{CO}_{3}^{2-}$$

**Figure 3:**
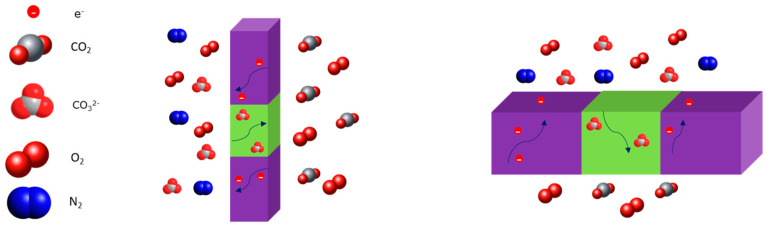
Schematic representation of a dual-phase metal-carbonate membrane. The arrows show the diffusion of species through the dual phase membrane.

On the feed side of the membrane, O_2_ combines with CO_2_ and electrons onto the molten carbonate surface and transforms into CO_3_^2−^ (Equation (5)). Carbonate ion is then transported through the molten carbonate phase and releases electrons, CO_2_, and O_2_ at the permeate side of the membrane (Equation (6)). Electrons will travel back to the feed side of the membrane, in the opposite direction of the carbonate ions, through the electron-conducting metal substrate guaranteeing the continuation of the permeation process (Figure 3). This permeation mechanism is called mixed electron-carbonate ion conduction (MECC).

A number of charge-transfer mechanisms and rate-limiting steps possibly involved in the electrochemical CO_2_ transport through the membrane have been investigated in the literature [20,21]. Based on the above literature, the ${CO}_{4}^{2-}$ scheme was concluded to be the best mechanism to describe CO_2_ transport through metal-carbonate membranes.

For the ${CO}_{4}^{2-}$ mechanism, the rate-limiting step can be described by Equation (8):

(8) $$\frac{1}{2}O_{2}+{CO}_{3}^{2-}\to {CO}_{4}^{2-}$$

The subsequent fast elementary charge-transfer steps that lead to the overall reaction when combined with the reaction are represented by Equations (9)–(11):

(9) $${CO}_{4}^{2-}+e^{-}={CO}_{3}^{2-}+{(O}^{-})$$

(10) $${(O}^{-})+e^{-}{\;=(O}^{2-})$$

(11) $${(O}^{2-})+{CO}_{2}={CO}_{3}^{2-}$$

**Figure 4:**
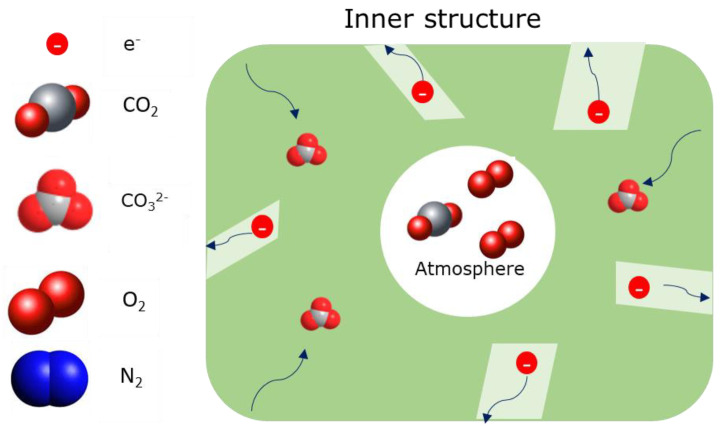
A representation of the charge-transfer model for the silver-carbonate membrane [17,18]. The arrows show the diffusion of species through the dual phase membrane.

In general, it was found that MC can provide an ideal surface for O_2_ molecule to stick on [22,23] and further allow O_2_ to chemically dissolve into MC as ${CO}_{4}^{2-}$. The formed ${CO}_{4}^{2-}$ can then migrate through the medium of MC according to the mechanism to the MC/metal surface [20], where it is reduced by electrons to ${CO}_{3}^{2-}$ and ${(O}^{-})$ (Equation (9)). This mechanism involves the breaking and reforming of O–${CO}_{3}^{2-}$ bond within and between ${CO}_{4}^{2-}$. Since the O in ${CO}_{4}^{2-}$ is weakly bonded, the migration of ${CO}_{4}^{2-}$ in MC is expected to be fast. The formed transient species ${(O}^{-}),$ as previously reported in the literature [23,24], are further reduced by electrons available at the MC/silver surface to ${(O}^{2-})$ (Equation (10)). The latter reacts with CO_2_ to form ${CO}_{3}^{2-}$ (Equation (11)) to complete the final transport of CO_2_ through the membrane (Figure 4).

The flux of gasses through the membrane is governed by the transport of carbonate ions in the molten phase and electronic charge carriers in the solid substrate under the Wagner-type transport theory electrochemical potential gradient [25]. The driving force for semi-reactions is the CO_2_ pressure gradient between the feed and permeate side of the membrane.

In this work, several eutectic alkalies and alkaline earth-based carbonate mixtures (ionic conducting phase) have been impregnated into porous stainless steel supports (electron-conducting phase) and tested. Porous substrates play a prominent role in the permeation process. Geometry and surface modification can increase the gas permeability and all of the substrate’s physical properties like thickness, acidity, wettability, pore volume and size, and tortuosity, and impact the permeability and efficiency of the membrane [21,26]. A porous stainless steel substrate (SS316) has been chosen for its good electron conductivity and especially for its mechanical properties [27,28].

## 2. Materials and Methods

### 2.1. Materials: Eutectic Mixtures

The physicochemical properties of the molten carbonates like electrical conductivity, density, surface tension, solubility, and impedance are different from the ones of their solid precursors [29,30,31]. The different properties will strongly affect the behavior and performance of the operating membranes.

The tested carbonate mixtures were selected taking into account their properties. First of all, the acidity/basicity properties strongly affect CO_2_ interaction with the membrane surface, and the utilization of more basic mixtures allows stronger interaction with the acid carbon dioxide, boosting the whole process. Normally, exhaust gasses are discharged at a temperature of around 420 °C [15,32]. Carbonate mixtures with appropriate melting temperatures have been then selected so that they can operate exploiting diesel engine exhaust gas temperature. High working temperatures must also be avoided in order not to have too high energy expenditure and in order to overcome the corrosion problems stainless steel suffers in the presence of lithium carbonates [33].

Alkali and alkaline-earth carbonate mixtures offering low melting temperature, favorable interactions with CO_2_, high thermal stability [33,34], and good physicochemical properties have been selected as the primary mixtures for investigation in this work. In addition, alkaline earth and rare earth element additives can modify the oxygen concentration in the melt by increasing basicity (improving the rate of O_2_ dissolution and CO_2_ flux).

For the purposes of this study, binary and ternary alkali and/or alkaline earth eutectic carbonate mixtures were studied in a temperature window of 300–650 °C in order for the adequate mixture for the desired application to be selected. The eutectic point of each carbonate mixture varies due to the carbonate mixture composition.

Based on the ternary phase diagram of Li_2_CO_3_/Na_2_CO_3_/K_2_CO_3_, the liquid-phase region of Li_2_CO_3_, K_2_CO_3_, and Na_2_CO_3_ system at 397–500 °C is from 40- to 65-mole ratio (%mol) of Li_2_CO_3_ [35]. With respect to this, trying to obtain the lowest eutectic points, binary and ternary systems that contain Li_2_CO_3_ at this range of mole ratio are being studied. These carbonate mixtures were chosen to be impregnated into an SS316 substrate in order for the desired reaction to commence at lower temperatures and achieve higher O_2_ permeability at lower temperatures.

Initially, the three main binary mixtures that were examined were all included in the temperature range of liquid phase 397–500 °C. These were Li_2_CO_3_/Na_2_CO_3_ = 52/48 (%mol), Li_2_CO_3_/K_2_CO_3_ = 52/48 (%mol), Li_2_CO_3_/K_2_CO_3_ = 62/38 (%mol), as shown in Table 1.

Ternary mixtures of Li_2_CO_3_/Na_2_CO_3_/K_2_CO_3_ were also studied based on these binary carbonate mixtures initially tested. More specifically, mixtures of Li_2_CO_3_/Na_2_CO_3_ = 52/48 (%mol) with the addition of 5, 15, and 25% K_2_CO_3_ (%mol) were analyzed for the same application in this publication, as shown in Table 2.

Furthermore, ternary mixtures of Li_2_CO_3_/Na_2_CO_3_/K_2_CO_3_ were studied based on the binary mixture Li_2_CO_3_/K_2_CO_3_ = 52/48 (%mol) with the addition of 5, 15, and 25% (%mol) Na_2_CO_3_ (%mol), as shown in Table 3.

Subsequently, ternary mixtures of Li_2_CO_3_/Na_2_CO_3_/K_2_CO_3_ were also studied based on the binary mixture Li_2_CO_3_/K_2_CO_3_ = 62/38 (%mol) with the addition of 5, 15, and 25% (%mol) Na_2_CO_3_ (%mol), as shown in Table 4.

In addition, the eutectic mixture with the lowest eutectic point of 397 °C, meaning Li_2_CO_3_/Na_2_CO_3_/K_2_CO_3_ = 43.5/31.5/25 (%mol) and the same mole ratio for all three carbonates, meaning Li_2_CO_3_/Na_2_CO_3_/K_2_CO_3_ = 33.3/33.3/33.3 (%mol) were examined, as shown in Table 5.

Finally, the binary Li_2_CO_3_/Na_2_CO_3_ *=* 52/48 (%mol) eutectic mixture was evaluated with additional CaCO_3_ (Ca ions) and BaCO_3_ (Ba ions) in various amounts: 0.5, 2.5, 5, and 10 (% mol), as shown in Table 6. According to the literature, the addition of a low molar fraction of Ca and Ba (0.5–10%) in the molten carbonate could improve oxygen solubility [33].

### 2.2. Dual-Phase Membrane Synthesis

**Figure 5:**
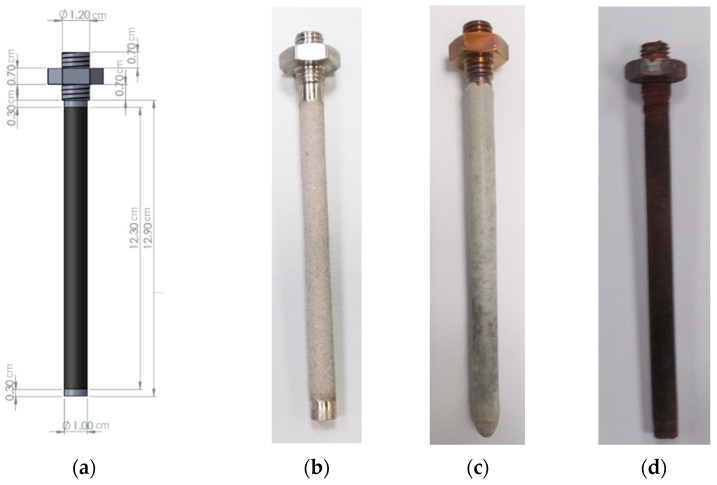
(**a**) Closed-end SS316 sintered mesh porous filters (unit: cm), (**b**) stainless steel filter substrate, (**c**) impregnated dual-phase membrane, and (**d**) calcinated dual-phase membrane.

In order to acquire dual-phase membranes, which consist of a metal porous phase and a carbonate phase, the impregnation of the SS316 filters took place in liquid-phase carbonate mixtures. Mixed electronic–ionic membranes were prepared by the direct dip impregnation of molten carbonate mixtures into porous 15 cm SS316, closed-end, powder porous tubes with porosity size of 1–10 μm (Figure 5a,b), with the method previously described and optimized [2].

**Figure 6:**
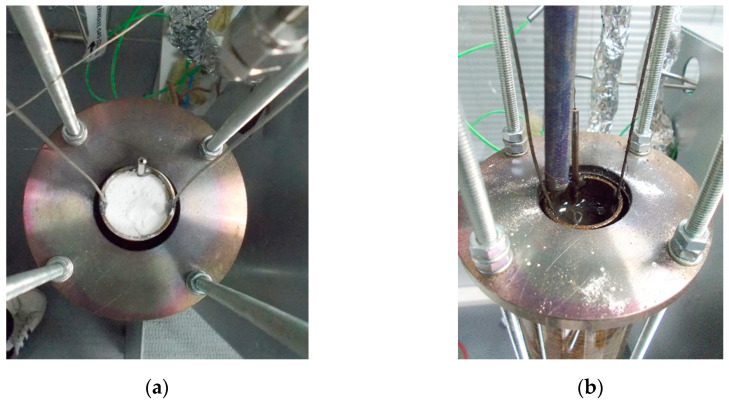
Synthesis procedure: (**a**) carbonate mixture placed in furnace; (**b**) molten carbonate mixture for SS filter impregnation.

In particular, the carbonate mixtures that were examined for the purposes of this publication were placed in a closed-end tube and subsequently in a custom-made quartz tube furnace (Figure 6a,b). The temperature was then elevated to the eutectic point until the phase was turned from solid to liquid and the impregnation was feasible (Figure 6b). Porous SS316 support was washed with acetone to avoid contamination and preheated above 500 °C to avoid oxidation due to the temperature difference between the carbonate mixture and the SS filter. High preheating temperature also grants higher carbonate infiltration into the SS tube [2]. The direct impregnation of stainless steel (SS316) filters into various molten carbonate mixtures was performed. Subsequently, after the impregnation, the impregnated membranes were left to dry in the air overnight (Figure 5c) and then calcinated for 1–2 h at a heating temperature range of 500–600 °C depending on the eutectic point of each carbonate mixture for the removal of the excess carbonate (Figure 5d). The above preparation procedure allows us to obtain stable, gas-tight dual-phase membranes at a temperature window of 400–550 °C. In order to examine the repeatability of the measurements, two membranes were prepared for each carbonate mixture tested.

### 2.3. CO_2_–O_2_ Permeation Through the Membrane: Process Simulation and Analysis

**Figure 7:**
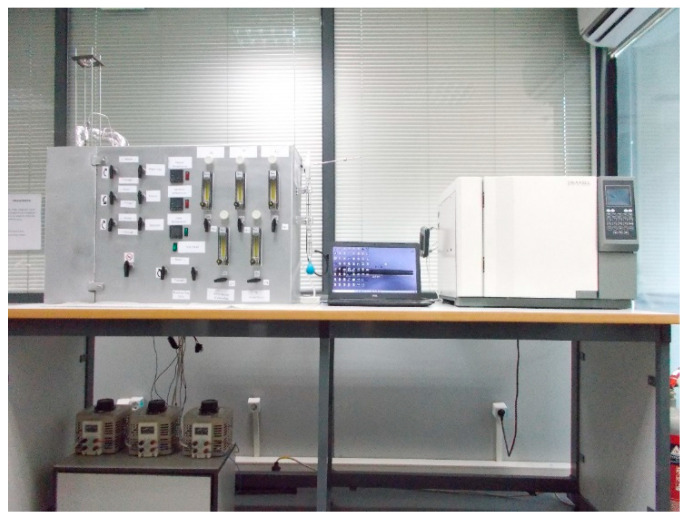
Synthetic gas bench of Oxygen Reduction System (ORS) and gas chromatography analyzer.

**Figure 8:**
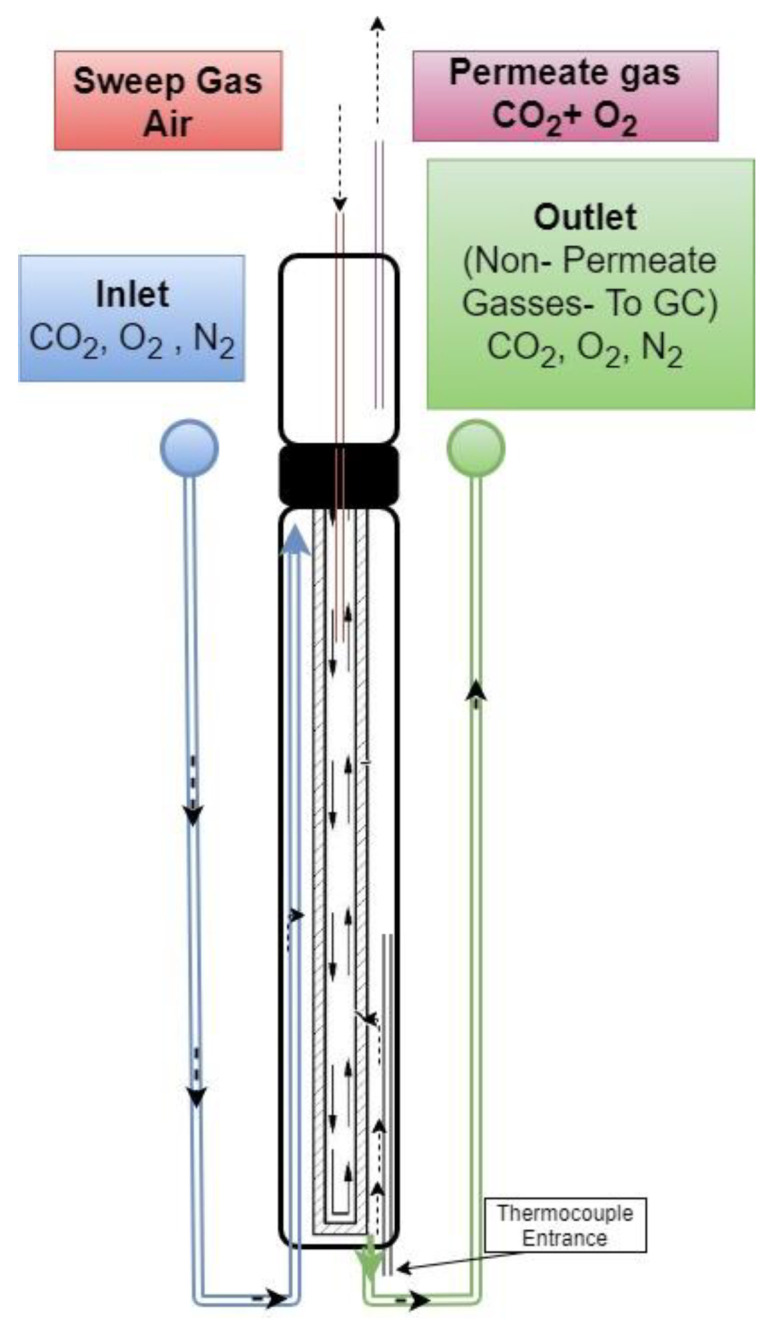
Schematic figure of the reactor of ORS.

A synthetic gas bench (SGB) was utilized in order to simulate the reaction that is under study (Equation (5)). A gas chromatograph (GC) was used for N_2_, CO_2_, and O_2_ gas analysis (Figure 7). This SGB of the ORS provides the ability to validate membrane permeability rates under various temperature, humidity, and gas mixture concentration conditions. The membrane of interest was placed into a cylinder reactor to be tested at the temperature range of 300–750 °C. The reactor (Figure 8) has two inlets (gas mixture input and sweep gas input) and two outlets (permeate gasses and non-permeate gasses). The reactor is designed to be fed with the inlet gas mixture through the lumen side, while CO_2_ and O_2_ will permeate across the membrane to the shell side and be released to the vent hood. The reactor is placed in a custom-made quartz tube furnace for the temperature to be elevated. The inlet gas mixture (12% CO_2_, 8% O_2_, and 80% N_2_, total flow rate = 300 cc/min) is regulated by the utilization of flow meters and the mixture is directed into the reactor containing the dual-phase membrane. As seen in Figure 8, the inlet gas comes into contact with the outer surface of the membrane. When the temperature reaches the eutectic point of each mixture, O_2_ combines with CO_2_ and electrons onto the molten carbonate surface and transforms into CO_3_^2−^ (Equation (5)). Carbonate ion is then transported through the molten carbonate phase and releases electrons, CO_2_, and O_2_ at the permeate side of the membrane (Equation (6)). The driving force for semi-reactions is the CO_2_ pressure gradient between the feed and permeate side of the membrane. The outlet gas that does not permeate through the surface of the membrane surface is analyzed by GC. It is also possible to supply a sweep gas from the shell side of the membrane to adjust the gas feed and composition of the CO_2_ and O_2_ mixture to the desired levels and to the desired flow. Permeation tests for each carbonate mixture were performed twice (two membranes were prepared for each carbonate mixture studied). A permeation test was also performed using a non-impregnated SS filter (SS316) as a baseline.

The SGB also allows the use of air as a sweep side at the inner side in order for the necessary partial pressure (p) gradient to be obtained and operate as the driving force for the enhancement of the permeation.

### 2.4. Dual-Phase Membranes for CO_2_ and O_2_ Separation Mechanism

The inlet gas of the reactor is brought in contact with the external surface of the membrane. The permeance of CO_2_ and O_2_ occurs through the surface of the carbonate membrane in mol·s^−1^·m^−2^. For the calculations, the external surface of the membrane with which the inlet gas is brought in contact in the reactor is used. The total reaction taking place in the dual-phase (molten carbonate/stainless steel phase) membrane is described by Equation (7). According to the reaction, the permeation rate of CO_2_ and O_2_ is calculated in mol·s^−1^·m^−2^, as follows:

The permeation rate of a gas *x* can be calculated as follows:

$$r_{x}=\frac{J\left(C_{x\left(in\right)}-C_{x\left(out\right)}\right)}{V_{m}A}$$

where

J (cm^3^∙min^−1^) = total flux of mixture;

C_x(in)_ (mol·L^−1^) = inlet concentration to the reactor of gas *x*;

C_x(out)_ (mol·L^−1^) = outlet concentration of the reactor of gas *x*;

V_m_ (cm^3^·mol^−1^) = molar volume at STP at room temperature and pressure 1 atm;

$A(m^{2})$ = external surface of the membrane.

$${r_{{CO}_{2}}\left(\frac{mol}{s\cdot m^{2}}\right)=r}_{{CO}_{3}^{2-}}=\frac{J\left(\frac{{cm}^{3}}{min}\right)\left(C_{{CO}_{2}\left(in\right)}\left(\%\right)-C_{{CO}_{2}\left(out\right)}\left(\%\right)\right)}{22,400\frac{{cm}^{3}}{mol}60\frac{s}{min}A(m^{2})}$$

and

$$r_{O_{2}}\left(\frac{mol}{s\cdot m^{2}}\right)=\frac{J\left(\frac{{cm}^{3}}{min}\right)\left(C_{O_{2}\left(in\right)}(\%)-C_{O_{2}\left(out\right)}\left(\%\right)\right)}{22,400\frac{{cm}^{3}}{mol}60\frac{s}{min}A(m^{2})}$$

where

J(cm^3^·min^−1^) = total flux of mixture;

$C_{\mathrm{gas}\left(in\right)}(\%)$= inlet concentration to the reactor of each gas;

$C_{\mathrm{gas}\left(out\right)}\left(\%\right)$= outlet concentration of the reactor (measured by GC);

22,400 (cm^3^·mol^−1^) = molar volume at STP at room temperature and pressure 1 atm [36];

$A(m^{2})$ = external surface of the membrane.

The symbols and notations which are used in this paper are summarized in Table A1 (Appendix A).

## 3. Results and Discussion

### 3.1. Binary Carbonate Mixtures

**Figure 9:**
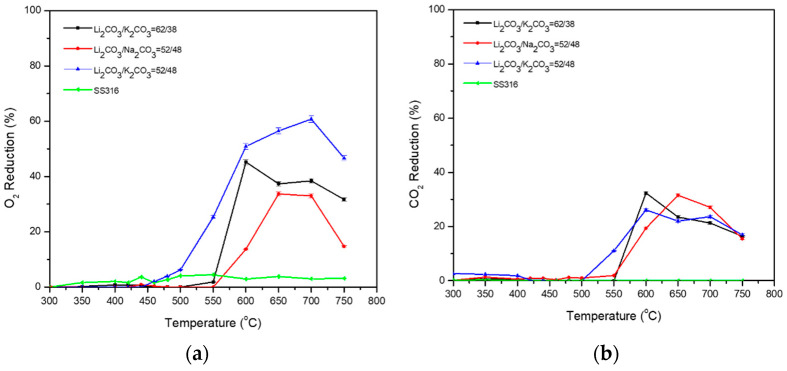
(**a**) O_2_ and (**b**) CO_2_ permeability of binary carbonate mixtures dual-phase membranes. A permeation test was also performed using a non-impregnated SS filter (SS316) as a baseline.

Initially, the binary carbonate mixtures were analyzed. According to Figure 9 and Table 7, the highest % O_2_ reduction (up to 60% at 700 °C) is achieved by the binary mixture of Li_2_CO_3_/K_2_CO_3_ = 52/48 (%mol), while lower %O_2_ reduction is exhibited by Li_2_CO_3_/Na_2_CO_3_ = 52/48 (%mol). In addition, Li_2_CO_3_/K_2_CO_3_ = 62/38 (%mol) shows a high % O_2_ reduction (35–45%) at temperatures higher than 600 °C. All the membranes exhibit 20–35% CO_2_ reduction at 600–750 °C temperature range, while Li_2_CO_3_/K_2_CO_3_ = 52/48 (%mol) shows better %CO_2_ reduction at 550 °C compared to the other two binary blends. All three binary mixtures were used as a basis to study ternary mixtures in which the third mixture will be added in 5, 15, and 25 (%mol). The negative values of the O_2_ permeation rate at ≤500 °C maybe could be attributed to parasitic reactions. At elevated temperatures, carbonates undergo thermal decomposition (M_2_CO_3_ → M_2_O + CO_2_, where M = Na, Li, and K) that forms O_2_ [37,38]. This reaction indicates that when carbonates are heated, they produce metal oxides and release CO_2_. However, under certain conditions (especially in the presence of other reactants or catalysts), O_2_ can be released as well. The released CO_2_ can then be reacted with other materials under specific conditions to produce O_2_. Also, metal oxides produced from the decomposition may further react with water vapor or other reducing agents at elevated temperatures to produce hydrogen gas and oxygen gas. The thermal treatment of Na_2_CO_3_/Li_2_CO_3_/K_2_CO_3_ eutectic molten carbonate mixture at 400–500 °C can lead to the production of oxygen gas through a combination of thermal decomposition and subsequent reforming reactions under appropriate conditions (M_2_CO_3_ → M^+^ + CO_2_ + O_2_ + e^−^, where M = Na, Li, and K).

### 3.2. Ternary Carbonate Mixtures

Subsequently, ternary mixtures of Li_2_CO_3_/Na_2_CO_3_/K_2_CO_3_ were also studied.

**Figure 10:**
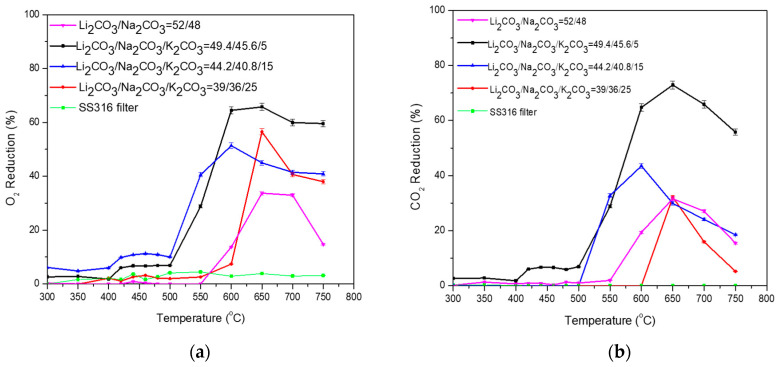
(**a**) O_2_ and (**b**) CO_2_ permeability of dual-phase membranes: Li_2_CO_3_/Na_2_CO_3_ = 52/48 (%mol) with 5, 15, and 25 (%mol) K_2_CO_3_. A permeation test was also performed using a non-impregnated SS filter (SS316) as a baseline.

Regarding the Li_2_CO_3_/Na_2_CO_3_ = 52/48 (%mol) with 5, 15, and 25 (%mol) K_2_CO_3_, the highest performance at temperature range 600–750 °C was exhibited for Li_2_CO_3_/Na_2_CO_3_/K_2_CO_3_ = 49.4/45.6/5 (%mol) as shown in Table 8 and Figure 10a. It is observed that the low percentage of 5 (%mol) K_2_CO_3_ resulted in the highest O_2_ reduction on the ternary mixture, showing the highest %O_2_ reduction (up to 65% at 600–650 °C) and %CO_2_ reduction (up to 70% at 650 °C), while the activation of the reaction was initiated at low temperature (500 °C). Increasing the percentage of K_2_CO_3_ further, the %O_2_ reduction was partially improved compared to the Li_2_CO_3_/Na_2_CO_3_ = 52/48 (%mol). Even though an increase of %O_2_ reduction is observed at the temperature range of 300–550 °C in the case of Li_2_CO_3_/Na_2_CO_3_/K_2_CO_3_ = 44.2/40.8/15 (%mol) compared to Li_2_CO_3_/Na_2_CO_3_/K_2_CO_3_ = 49.4/45.6/5 (%mol), a decrease of the %O_2_ reduction is recorded at higher temperatures. Compared to the Li_2_CO_3_/Na_2_CO_3_ = 52/48 (%mol), the %CO_2_ reduction is improved in the case of 15% K_2_CO_3_ at a temperature range of 550–600 °C, but it is lower in the case of 25% K_2_CO_3._

Some of these mixtures showed a significant formation of CO_2_ at low temperatures until the desirable reaction was activated, as negative values appear in Figure 10b. This could be attributed to the carbonate salt mixture’s composition to produce CO_2_. Τhe thermal stability of the binary salt Li_2_CO_3_/Na_2_CO_3_ (42/58 wt%) as a phase change material for thermal energy storage through thermal decomposition analysis in different environments and thermal cycling was investigated by Jiang et al. [37]. According to Jiang et al., it was noticed that there was a weight loss in N_2_ at the melting point in the range of 500 °C. The evolving gaseous species of CO_2_ in N_2_ was detected at 500 °C by a Simultaneous Thermal Analyzer (STA/TG-MS), which slightly increased with increasing temperatures. This confirms that CO_2_ was produced in the heating process at temperatures above 500 °C. No other evolving gaseous species were observed. The weight loss of Li_2_CO_3_/Na_2_CO_3_ probably results from the salt’s composition to produce CO_2_ as shown in the following reactions (Equations (12) and (13)) [37]:

Na_2_CO_3_ = Na_2_O + CO_2_ (g) (12)

Li_2_CO_3_ = Li_2_O + CO_2_ (g) (13)

As previously reported in the literature [34], under the presence of air, the gaseous CO_2_ evolution from the salt is observed to commence at 530 °C. The onset of decomposition of Li_2_CO_3_/Na_2_CO_3_/K_2_CO_3_ (32.1/33.4/34.5 wt%) was detected by a Differential Scanning Calorimetry (DSC) analysis at 601 °C and the rapid rate of weight loss determined by a thermogravimetric (TG) analysis at 673 °C [34].

**Figure 11:**
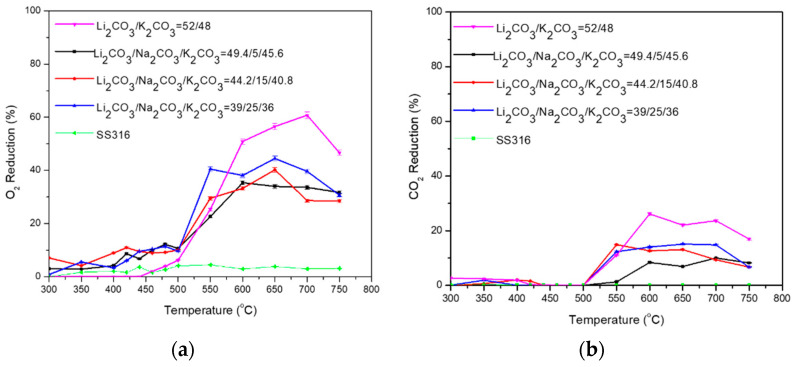
(**a**) O_2_ and (**b**) CO_2_ permeability of dual-phase membranes: Li_2_CO_3_/K_2_CO_3_ = 52/48 (%mol) with 5, 15, and 25 (%mol) Na_2_CO_3_. A permeation test was also performed using a non-impregnated SS filter (SS316) as a baseline.

According to Table 9 and Figure 11a,b, for the Li_2_CO_3_/K_2_CO_3_ = 52/48 (%mol) with 5, 15, and 25 (%mol) Na_2_CO_3_, the highest %O_2_ and %CO_2_ reduction is achieved by the binary Li_2_CO_3_/K_2_CO_3_ = 52/48 (%mol) mixture at temperatures >600 °C. The reaction is activated around 500 °C for all the mixtures (in accordance with the temperature window phase change in Table 3), reaching a maximum at approximately 550–650 °C. The permeability is not significantly affected according to the added quantity of Na_2_CO_3_. It is observed that the high percentage of 25 (%mol) Na_2_CO_3_ had the best impact on the ternary mixture, showing the highest %O_2_ reduction (up to 40%) at 550 °C compared to the other ternary mixtures and Li_2_CO_3_/K_2_CO_3_ = 52/48 (%mol) mixture. The permeability is not improved by the addition of Na_2_CO_3_ and the results are similarly lower than the binary mixture at temperatures higher than 600 °C.

**Figure 12:**
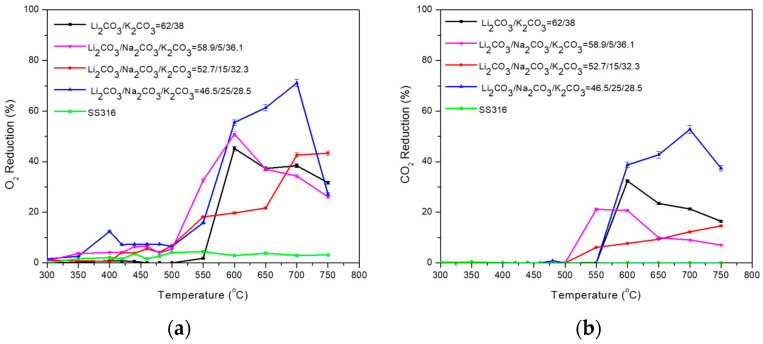
(**a**) O_2_ and (**b**) CO_2_ permeability of dual-phase membranes: Li_2_CO_3_/K_2_CO_3_ = 62/38 (%mol) with 5, 15, and 25 (%mol) Na_2_CO_3_. A permeation test was also performed using a non-impregnated SS filter (SS316) as a baseline.

Subsequently, the Li_2_CO_3_/K_2_CO_3_ = 62/38 (%mol) with 5, 15, and 25% Na_2_CO_3_, were examined (Table 10, Figure 12). The addition of Na_2_CO_3_ enhances %O_2_ reduction at 550 °C. When the temperature is further increased, Li_2_CO_3_/Na_2_CO_3_/K_2_CO_3_ = 58.9/5/36.1 (%mol) shows similar performance (regarding O_2_% reduction) with Li_2_CO_3_/K_2_CO_3_ = 62/38 (%mol). The addition of 5% and 15% Na_2_CO_3_ enhances %CO_2_ reduction at 550 °C, but by increasing the temperature further, the performance is lower compared to Li_2_CO_3_/K_2_CO_3_ = 62/38 (%mol). The addition of 25% (%mol) Na_2_CO_3_ has a good impact on the permeability and subsequently on %O_2_ (55–70%) and % CO_2_ (40–55%) reduction at high temperatures (>650 °C). It is observed that the high percentage of 25 (%mol) Na_2_CO_3_ on the ternary blend results in the highest %O_2_ and %CO_2_ reduction at temperatures higher than 600 °C compared to the other ternary mixtures and Li_2_CO_3_/K_2_CO_3_ = 62/38 (%mol) mixture.

**Figure 13:**
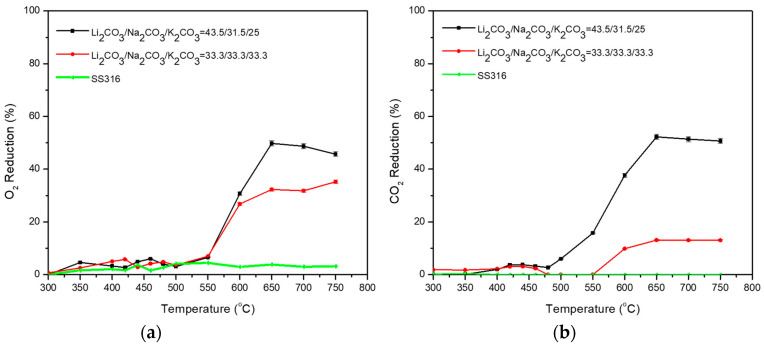
(**a**) O_2_ and (**b**) CO_2_ permeability of dual-phase membranes Li_2_CO_3_/Na_2_CO_3_/K_2_CO_3_ = 43.5/31.5/25 (%mol) and Li_2_CO_3_/Na_2_CO_3_/K_2_CO_3_ = 33.3/33.3/33.3 (%mol). A permeation test was also performed using a non-impregnated SS filter (SS316) as a baseline.

The eutectic mixture Li_2_CO_3_/Na_2_CO_3_/K_2_CO_3_ = 43.5/31.5/25 (%mol) was also examined as well as the same composition of the three carbonates Li_2_CO_3_/Na_2_CO_3_/K_2_CO_3_ = 33.3/33.3/33.3 (%mol), Table 11. The eutectic mixture exhibited a shoulder at the O_2_ raising permeation rate at the temperature window 450–500 °C (Figure 13), which can be attributed to the state change in the carbonate mixture due to the lower eutectic point. The carbonate mixture is liquified, and the permeation rate (*r*_*O*2_) increases in relation to the permeation rate in the solid state of the mixture.

Regarding the ternary mixtures, Li_2_CO_3_/Na_2_CO_3_/K_2_CO_3_ = 44.2/40.8/15 (%mol) and Li_2_CO_3_/Na_2_CO_3_/K_2_CO_3_ = 39/25/36 (%mol) mixtures have the highest performance at 550 °C compared to the other blends (40% O_2_ reduction). The reduction of O_2_ in the case of Li_2_CO_3_/Na_2_CO_3_/K_2_CO_3_ = 49.4/45.6/5 (%mol) and Li_2_CO_3_/Na_2_CO_3_/K_2_CO_3_ = 46.5/25/28.5 (%mol) mixtures gives an ideal performance as high as 65% O_2_ at 600–650 °C and 70% O_2_ at 700 °C, respectively.

**Figure 14:**
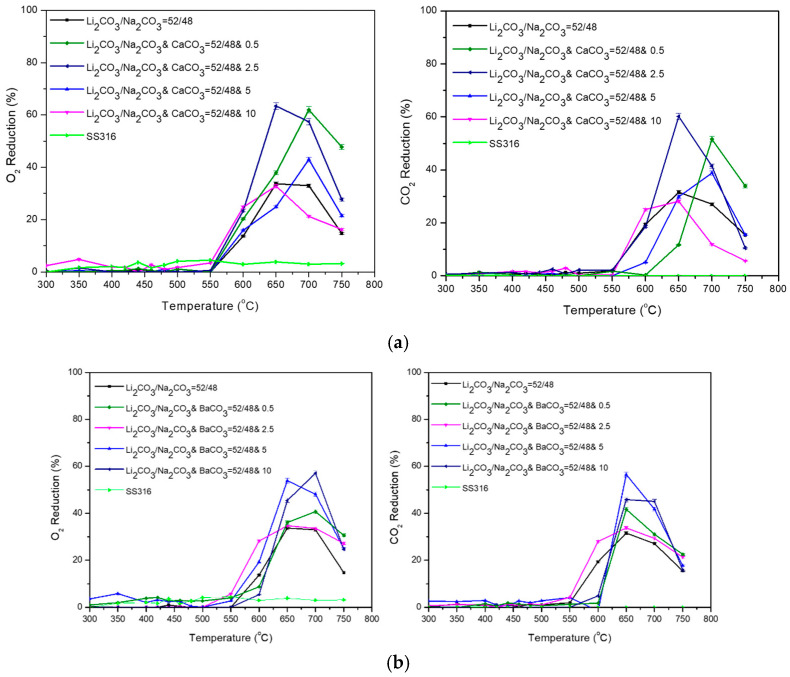
Permeability of dual-phase membranes: (**a**) Li_2_CO_3_/Na_2_CO_3_ and CaCO_3_ = 52/48 (%mol) and 0.5, 2.5, 5, or 10% mol and (**b**) Li_2_CO_3_/Na_2_CO_3_ and BaCO_3_ = 52/48 (%mol) and 0.5, 2.5, 5, or 10% mol. A permeation test was also performed using a non-impregnated SS filter (SS316) as a baseline.

Finally, the binary Li_2_CO_3_/Na_2_CO_3_ = 52/48 (%mol) mixture was studied with additional CaCO_3_ (Ca ions) and BaCO_3_ (Ba ions) in various compositions (Figure 14). The mixture was used as a basis due to lower %O_2_ and %CO_2_ reduction compared to the other two binary mixtures. According to the experimental results, at temperatures >650%, the %O_2_ and %CO_2_ reduction improved by the addition of Ca ion or Ba ions as compared to pure Li_2_CO_3_/Na_2_CO_3_ = 52/48 (%mol) mixture, except Li_2_CO_3_/Na_2_CO_3_ = 52/48 and 10% (%mol) CaCO_3_. This maybe could be attributed to the enhancement of oxygen dissolution in the carbonate melt and thus the permeability of O_2_ and CO_2_ as well [34]. The results indicate that the reduction of O_2_ and CO_2_ reaches a maximum of 650–700 °C. The reduction of O_2_ in the presence of these cations gives an ideal performance as high as 65% O_2_ reduction for Li_2_CO_3_/Na_2_CO_3_ and CaCO_3_ = 52/48 and 2.5 (%mol) and 55% O_2_ reduction for Li_2_CO_3_/Na_2_CO_3_ and BaCO_3_ = 52/48 and 5 (%mol) at 650 °C.

### 3.3. O_2_/CO_2_ Flux Ratio

**Figure 15:**
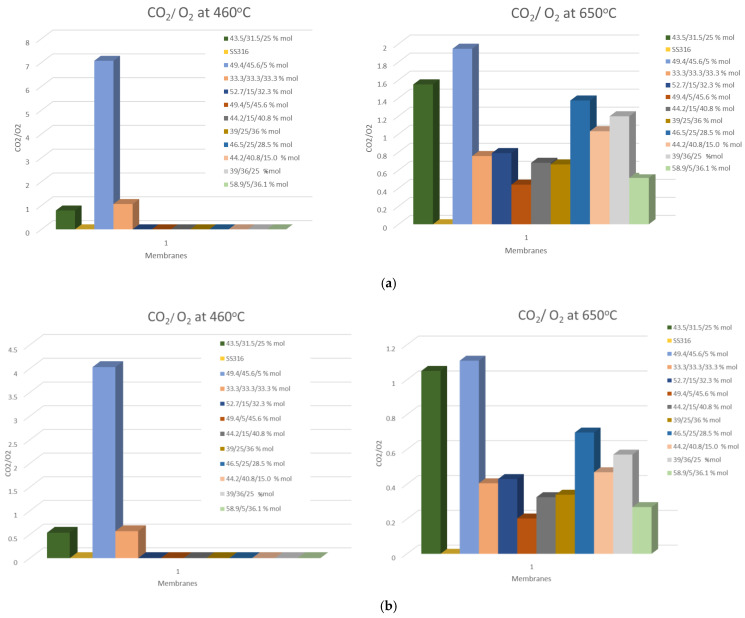
(**a**) J_CO2_/J_O2_ permeation rate ratio and (**b**) %CO_2_/%O_2_ reduction rate ratio of several Li_2_CO_3_/Na_2_CO_3_/K_2_CO_3_ membranes developed in this study.

According to the literature, in MECC membranes, the ratio between J_O2_ and J_CO2_ is commonly equal to two, as theoretically expected from the overall electrochemical reaction of O_2_/CO_2_ transport through the membrane (CO_2_ + ½ O_2_ + 2e^−^ → CO_3_^2−^) [20,29]. Based on a recent work [39], Atomic Layer Deposition (ALD) Al_2_O_3_-coated Ag-MC membranes show a J_O2_:J_CO2_ ratio of about 1.5:1 due to the increased wettability between Ag and molten carbonate and improved O_2_ reactivity with CO_3_^2−^ to form CO_4_^2−^. It is known that the solubility of CO_2_ in molten carbonates is much higher than that of O_2_. As a result, the oxygen dissolution of molten carbonate plays an important role in the permeation mechanism. The incorporation of O_2_ into a molten carbonate is closely related to the rate-limiting step changes, which could affect the possible dominating mechanism of the transport. In Figure 15, the J_CO2_:J_O2_ ratio at 460 and 650 °C of the eutectic Li_2_CO_3_/Na_2_CO_3_/K_2_CO_3_ mixtures developed in this study is presented. The deviations could be attributed to the variation in O_2_ reactivity with CO_3_^2−^ (and also O_2_ solubility) with the change in molten carbonate composition.

To conclude, the % reduction of O_2_ gas was examined for multiple binary and ternary alkali and alkaline earth metal-carbonate mixtures dual-phase membranes as a function of temperature in the range of 300–750 °C. In order to implement the use of carbonate membranes in HDV diesel engines, carbonate mixtures were chosen to take into account the temperature of diesel engine exhaust gasses. The temperature of the gasses varies with the load, and at 100% load, they are ejected in a range of temperatures between 500 °C and 700 °C. The operating temperature of the membranes is above the melting temperature of the selected carbonate mixtures. For all the carbonate mixtures, it is observed that dissolution and diffusion in the molten carbonate phase of gas molecules are facilitated and enhanced by temperature elevation. More specifically, the initial gas permeance of O_2_ is relatively low and increases with rising temperature. After the carbonate mixture changes state, upon reaching the eutectic point and passing from the solid state to complete liquefaction, the permeation rate increases rapidly. The results indicate that the permeance of O_2_ and CO_2_ reaches a maximum of 600–700 °C.

As for the gas permeance of CO_2_, the general trend is the same, as the permeance is low at lower temperatures and increases upon reaching and passing the eutectic point. According to the results, in some cases, the carbonate mixtures exhibit an augmentation of CO_2_ concentration at low temperatures (around 500 °C), which is attributed to the decomposition of the carbonates. When the permeation commences, the reaction of the permeability overcomes the decomposition and CO_2_ permeates through the membrane. The ternary mixture Li_2_CO_3_/Na_2_CO_3_/K_2_CO_3_ = 49.4/45.6/5 (%mol) shows the highest performance for both O_2_ and CO_2_ reduction at 600 °C. The reduction of CO_2_ in the presence of these cations’ composition gives an ideal performance as high as 60% at 600 °C and 70% at 650 °C. The reduction of CO_2_ into Li_2_CO_3_/Na_2_CO_3_ = 52/48 (%mol) mixture increases with the addition of Ca and Ba ions compared to the pure mixture. The permeation rate of CO_2_ was not included in the tables of the manuscript, since the main focus of the work is the application of dual-phase mixed electron-carbonate ion conducting (MECC) membranes in HDVs (diesel engine vehicles) for O_2_ removal from the exhaust gas stream (O_2_ has the dominant role in the specific application).

The observed decrease in O_2_ and CO_2_ permeation rates at higher temperatures (>700 °C) has been reported in previous studies [29], and can be attributed to several factors related to the material and transport properties of MECC membranes. Higher temperatures generally increase molecular mobility, which can further enhance gas permeation rates. However, it is essential to balance temperature effects with potential changes in membrane structure due to exposure to high temperatures. At >700 °C, there may be phase transitions within the eutectic molten carbonates’ mixture, leading to changes in the microstructure and porosity of the membrane. Induced transitions between different structural forms or phases within the membrane material could either enhance or inhibit CO_2_ transport depending on specific interactions. For instance, if the membrane undergoes a transition to a less conductive phase or if sintering effects reduce the available porosity, this could result in a decrease in the effective permeation rate. It is also reported in the literature [29] that at temperatures ≥650 °C, interfacial stainless steel–molten carbonate mixtures (Li_2_CO_3_/Na_2_CO_3_/K_2_CO_3_) reactions lead to the formation of Li–Fe–O phases (LiFe_5_O_8_ and LiFeO_2_) on the surface of the support (structural changes). As the electronic conductivity of these oxide phases is very low, the electron transport pathway through the support is lost and the performance of the MECC membrane is affected (reduction in CO_2_ permeation). In addition, at elevated temperatures (>700 °C), the ionic conduction might be reduced due to the depletion of certain ionic species within the membrane, potentially limiting the availability of mobile species (such as oxygen ions or carbonate ions) for permeation. Furthermore, at high temperatures, side reactions, such as the decomposition of CO_2_ into CO and O_2_, might compete with the expected transport process. These side reactions (competing reactions) could also affect the efficiency of the transport mechanism, leading to a decrease in the overall permeation rates of the MECC membranes. This behavior is consistent with the findings in other dual-phase MECC membranes, where similar trends have been reported. These observations align with our findings, supporting the idea that both the material characteristics and temperature-induced structural changes contribute to the decrease in the permeation rates of MECC at higher temperatures.

The investigated membrane system combines the unique combinations of metal-carbonates and electronic conducting phases, which may result in different ionic and electronic conductivity profiles compared to the membranes explored in the literature [17,26,29]. The use of molten carbonate salts as an ionic conducting phase of dual-phase MECC membranes allows the production of highly permeable membranes (high CO_2_ and O_2_ solubility) with theoretically infinite selectivity [17,26]. On the other hand, Mixed Ionic–Electronic Conducting membranes (MIEC) primarily facilitate both ionic and electronic conduction, but may not incorporate carbonate phases. Common materials include perovskites or other oxide-based compounds that exhibit good ionic conductivity alongside electron transport capabilities. While MIEC membranes demonstrate good oxygen permeation rates, they may not reach the same levels as some optimized MECCs due to differences in material properties and structural configurations. Mixed electron-carbonate ion conducting membranes can achieve relatively high O_2_ permeation rates due to their dual conduction pathway, which allows for efficient transport mechanisms. In MECC membranes, the O_2_ permeation rate is strongly affected by melt basicity, while properties (such as viscosity) and the proper wetting of molten carbonates in the support are vital for high-performance MECC membrane development. Furthermore, the CO_2_ permeation mechanism can be varied with the change in molten carbonate composition. High permeation rates of CO_2_ and O_2_ can be achieved using the investigated MECC membranes compared to the ones reported in the literature due to the synergistic effect of membrane structure and composition, and microstructure features (such as phase distribution and porosity). The specific selection of materials in the investigated membrane system (composition of the molten carbonate phase, type of SS filter) is aimed at optimizing both the ionic conductivity and the electronic conductivity (efficient electrochemical reactions), which may differ from other systems in the literature for different applications (different operational conditions) that focus more on either ionic or electronic conductivity separately.

Compared with the literature findings, during the presented study, the simultaneous separation of O_2_ and CO_2_ from the flue gas stream can be achieved without the need to supply electrical energy through external lead wires (no external electrical power is supplied or stored). With the presence of O_2_, CO_2_ undergoes a charge transfer reaction on the upstream membrane surface to form a charged ${CO}_{3}^{2-}$. The charged carbonaceous species diffuse toward the other side of the membrane and are converted to the molecular CO_2_ and O_2_ in a reverse reaction on the downstream membrane surface. During the process, the electrons move in the opposite direction through the metal support. The electron transports back, through the metal phase, toward the upstream membrane surface (electrochemical transport membrane/selective electrochemical CO_2_ separation membrane). The electrochemical gradients of CO_3_^2−^ and e^−^ exist in the opposite direction across the membrane. The outward diffusion of O_2_ is driven by the chemical potential gradient of both CO_2_ and O_2_. The results of this study are very promising for the application of O_2_ reduction in diesel HDVs.

Recent publications focus on the effect of steam on the permeation of CO_2_ in dual-phase mixed electronic-carbonate ion conducting membranes [40]. Steam can enhance the permeation of CO_2_ of dual-phase mixed electron-carbonate ion conducting membranes due to hydrophilic interactions (the formation of hydroxide ions in molten carbonate phase) and temperature-induced mobility (increase and alterations in molten carbonate phase behavior). A more hydrophilic environment within the MECC membrane structure may facilitate better solubility and diffusion pathways for CO_2_. Understanding these interactions, but also potential long-term stability concerns, is critical for optimizing dual-phase mixed electron-carbonate ion conducting membranes for applications involving CO_2_ separation and capture. As several factors can affect the gas permeability of dual-phase mixed electron-carbonate ion conducting membranes, in the first tests, different membrane compositions have been examined under the simulated emission concentration of a diesel engine in the absence of steam (humidity) to identify the effect of carbonates on permeation rate and for better understanding and validation of the results. An introduction study was performed in this manuscript to explore the effect of gas species (CO_2_, O_2_, and N_2_) on the MECC membrane’s permeation properties for O_2_ removal from the exhaust gas stream of HDVs. Our study aimed to isolate and understand this particular permeation behavior of O_2_ in the context of CO_2_ and N_2_ using different molten carbonate compositions that are impregnated into an SS filter. Even though steam is a relevant factor in diesel engine exhaust and could be a perspective of future work, the incorporation of humidity in the simulated measurements was not in the main scope of the current study (industrial point of view). Subsequently, stability tests and aging procedures under various parameters that simulate the HDVs diesel exhaust gas system could be performed to assure the efficiency over time or further need for coating.

As several factors can influence the gas permeability of dual-phase MECC membranes (complex reaction mechanism), the current study is focused on the effect of gas species like CO_2_, O_2_, and N_2_ on different dual-phase mixed electron-carbonate ion conducting membrane compositions to identify the most promising membrane’s configuration (high permeation rate) for the efficient removal of the excess of O_2_ from the exhaust gas mixture of an HDV (the successful reduction of NO_x_ to less harmful gasses on the reduction catalyst). The approach of this work is from an industrial point of view. In this manuscript, membranes’ performance in terms of O_2_ removal in HDV applications has been demonstrated. This allowed us to establish the initial functional potential of the material. As dual-phase MECC membranes may result in higher permeation rates, several carbonates’ compositions have been studied in combination with SS for the removal of the excess of O_2_ from exhaust gas stream of HDVs. Based on the requirements of the materials for this specific application (high flux, robustness towards vibrations, and thermal stability), high surface area SS filters are carefully designed and developed. Pore size distribution (porosity) is the main specification for the development of high-performance MECC membranes (high O_2_ permeation rates) for HDV diesel applications.

As the scope of the manuscript is to study the application of different carbonate mixtures impregnated in a porous stainless steel support for the O_2_ removal from the exhaust gas stream of diesel vehicles (industrial application and under specific conditions), the physicochemical characterization of the MECC membranes was not be emphasized. An X-Ray Fluorescence (XRF) analysis of the membranes, which provides valuable insights into the elemental composition and distribution within the membrane, has also been conducted. The uncoated SS316 filter composition is in accordance with the literature findings (high-grade austenitic stainless steel, elemental composition by weight percentage for the elements: Fe ~ 67%, Cr ~ 17%, Ni ~ 12%, Mo ~ 2%, Mn ~ 1%, Si < 1%, and traces of Co). The relative intensities of Li, Na, and K elements depend on the thickness of the coating and the concentration of each element. In the case of Li_2_CO_3_/Na_2_CO_3_/K_2_CO_3_ = 49.4/45.6/5 (%mol) mixture (one of the best-performing membranes of this study), the XRF analysis results are in accordance with the nominal composition considering the nominal molar fractions (the overall weight percentage of each element in the coating: 9.3% Li, 19.8% Na and 2.8% K). Physicochemical property data for further membrane formulation optimization based on the final specifications of the HDV or additional structural characterization (e.g., phase structure and morphology of the membranes, thermal stability, mechanical properties) could be perspectives of future work to establish a clearer structure-performance relationship.

## 4. Conclusions

In this work, a dense membrane that consists of two phases, where each has a separate role, for oxygen removal in the exhaust gas of lean-burn engines is described. No external electrodes and connectors are required in this dual-phase membrane. The novelty of this membrane is that the non-spontaneous uphill diffusion of O_2_ from the flue gas to the high O_2_ concentration side (atmosphere) can be achieved without any external electric power. The permeated gas stream contains both CO_2_ and O_2_ at a given concentration range.

Several eutectic alkalies and/or alkaline earth-based molten carbonate mixtures (ionic conducting phase) impregnated in a porous stainless steel (SS316) support (electron-conducting phase) were synthesized and tested. Experimentally, for all the carbonate mixtures, it is observed that the permeation of CO_2_ and O_2_ is enhanced by temperature elevation. Furthermore, most samples exhibit an augmentation of CO_2_ concentration at low temperatures, which is attributed to the decomposition of the carbonates, which is subsequently overcome when the permeation commences and CO_2_ permeates through the membrane. This study shows that there are carbonate mixtures that could be utilized for diesel HDV applications, allowing them to meet the emission and air quality EURO VI standards. According to the results, Li_2_CO_3_/Na_2_CO_3_/K_2_CO_3_ = 44.2/40.8/15 (%mol) and Li_2_CO_3_/Na_2_CO_3_/K_2_CO_3_ = 39/25/36 (%mol) exhibit high O_2_ permeability (40% O_2_ reduction) at 550 °C). The permeation rate of Li_2_CO_3_/Na_2_CO_3_/K_2_CO_3_ = 49.4/45.6/5 (%mol) mixture increases at low temperatures (approximately 300–500 °C) reaching up to 0.3 mol·s^−1^·m^−2^ (65% O_2_ reduction at 600–650 °C). The temperature window coincides with the operational exhaust temperature of refuse HDVs, making it suitable for the application of O_2_ reduction in diesel HDVs. The permeate oxygen can be either released into the atmosphere or used onboard to improve the efficiency of the engine utilizing the high O_2_ content.

However, further study could be performed (e.g., the addition of different alkaline earth metals, operational conditions of a membrane, etc.) to ensure the proper operation of the system at the exhaust of an HDV. Carbonate blends using additives (e.g., NaOH, Sr, Cs, Rb carbonate, and rare earth oxides) could be explored in an attempt to decrease the melting point and improve further the ionic conductivity of eutectic carbonate mixtures. Furthermore, stability tests could be a perspective of future work to ensure efficient operation over time and to facilitate whether there is a further need for coating.

## 5. Patents

ORS is IP protected at the National and European levels (Greek patent granted: GR10094788 (9 February 2018.), European Patent Published: GR3542887A1 (25 September 2019)).

## Figures and Tables

**Table 1:** Binary carbonate mixtures.

Carbonate Mixture	Melt Composition (%mol)	Experimental Temperature Window of Phase Change from Solid to Liquid (°C)
Li_2_CO_3_/Na_2_CO_3_	52/48	500–550
Li_2_CO_3_/K_2_CO_3_	52/48	480–520
Li_2_CO_3_/K_2_CO_3_	62/38	500–530

**Table 2:** Ternary carbonate mixtures, with binary Li_2_CO_3_/Na_2_CO_3_ = 52/48 (%mol) as basis.

Carbonate Mixture	Melt Composition (%mol)	Experimental Temperature Window of Phase Change from Solid to Liquid (°C)
Li_2_CO_3_/Na_2_CO_3_	52/48	500–550
Li_2_CO_3_/Na_2_CO_3_/K_2_CO_3_	49.4/45.6/5	500–530
Li_2_CO_3_/Na_2_CO_3_/K_2_CO_3_	44.2/40.8/15	500–530
Li_2_CO_3_/Na_2_CO_3_/K_2_CO_3_	39/36/25	450–520

**Table 3:** Ternary carbonate mixtures, with binary Li_2_CO_3_/K_2_CO_3_ = 52/48 (%mol) as basis.

Carbonate Mixture	Melt Composition (%mol)	Experimental Temperature Window of Phase Change from Solid to Liquid (°C)
Li_2_CO_3_/K_2_CO_3_	52/48	480–520
Li_2_CO_3_/Na_2_CO_3_/K_2_CO_3_	49.4/5/45.6	480–530
Li_2_CO_3_/Na_2_CO_3_/K_2_CO_3_	44.2/15/40.8	470–520
Li_2_CO_3_/Na_2_CO_3_/K_2_CO_3_	39/25/36	450–500

**Table 4:** Ternary carbonate mixtures, with binary Li_2_CO_3_/K_2_CO_3_ = 62/38 (%mol) as basis.

Carbonate Mixture	Melt Composition (%mol)	Experimental Temperature Window of Phase Change from Solid to Liquid (°C)
Li_2_CO_3_/K_2_CO_3_	62/38	500–530
Li_2_CO_3_/Na_2_CO_3_/K_2_CO_3_	58.9/5/36.1	500–550
Li_2_CO_3_/Na_2_CO_3_/K_2_CO_3_	52.7/15/32.3	480–530
Li_2_CO_3_/Na_2_CO_3_/K_2_CO_3_	46.5/25/28.5	470–520

**Table 5:** Eutectic ternary mixtures and equal mole ratio.

Carbonate Mixture	Melt Composition (%mol)	Experimental Temperature Window of Phase Change from Solid to Liquid (°C)
Li_2_CO_3_/Na_2_CO_3_/K_2_CO_3_	43.5/31.5/25	430–500
Li_2_CO_3_/Na_2_CO_3_/K_2_CO_3_	33.3/33.3/33.3	500–550

**Table 6:** Eutectic ternary mixtures, with binary Li_2_CO_3_/Na_2_CO_3_ = 52/48 (%mol) as basis and equal composition.

Carbonate Mixture	Melt Composition (%mol)
Li_2_CO_3_/Na_2_CO_3_/CaCO_3_	52/48/0.5
Li_2_CO_3_/Na_2_CO_3_/CaCO_3_	52/48/2.5
Li_2_CO_3_/Na_2_CO_3_/CaCO_3_	52/48/5
Li_2_CO_3_/Na_2_CO_3_/CaCO_3_	52/48/10
Li_2_CO_3_/Na_2_CO_3_/BaCO_3_	52/48/0.5
Li_2_CO_3_/Na_2_CO_3_/BaCO_3_	52/48/2.5
Li_2_CO_3_/Na_2_CO_3_/BaCO_3_	52/48/5
Li_2_CO_3_/Na_2_CO_3_/BaCO_3_	52/48/10

**Table 7:** Oxygen permeability rate (mol·s^−1^·m^−2^) of binary mixtures for temperature window 500–650 °C.

Carbonate Mixture	Melt Composition (%mol)	500 °C	550 °C	600 °C	650 °C
		O_2_ Permeation Rate (mol·s^−1^·m^−2^)			
Li_2_CO_3_/Na_2_CO_3_	52/48	−0.012	−0.005	0.059	0.146
Li_2_CO_3_/K_2_CO_3_	52/48	0.024	0.099	0.198	0.220
Li_2_CO_3_/K_2_CO_3_	62/38	−0.001	0.009	0.212	0.175

**Table 8:** Oxygen permeability rate (mol·s^−1^·m^−2^) of ternary mixtures with the binary mixture Li_2_CO_3_/Na_2_CO_3_ = 52/48 (%mol) as the basis for the temperature window of 500–650 °C.

Carbonate Mixture	Melt Composition (%mol)	500 °C	550 °C	600 °C	650 °C
		O_2_ Permeation Rate (mol·s^−1^·m^−2^)			
Li_2_CO_3_/Na_2_CO_3_	52/48	−0.012	−0.005	0.059	0.146
Li_2_CO_3_/Na_2_CO_3_/K_2_CO_3_	49.4/45.6/5	0.034	0.142	0.317	0.324
Li_2_CO_3_/Na_2_CO_3_/K_2_CO_3_	44.2/40.8/15	0.061	0.201	0.254	0.217
Li_2_CO_3_/Na_2_CO_3_/K_2_CO_3_	39/36/25	0.008	0.011	0.031	0.239

**Table 9:** Oxygen permeability rate (mol·s^−1^·m^−2^) of ternary mixtures with the binary mixture Li_2_CO_3_/K_2_CO_3_ = 52/48 (%mol) as the basis for the temperature window of 500–650 °C.

Carbonates Mixture	Melt Composition (%mol)	500 °C	550 °C	600 °C	650 °C
		O_2_ Permeation Rate (mol·s^−1^·m^−2^)			
Li_2_CO_3_/K_2_CO_3_	52/48	0.024	0.099	0.198	0.220
Li_2_CO_3_/Na_2_CO_3_/K_2_CO_3_	49.4/5/45.6	0.051	0.108	0.168	0.162
Li_2_CO_3_/Na_2_CO_3_/K_2_CO_3_	44.2/15/40.8	0.049	0.143	0.161	0.194
Li_2_CO_3_/Na_2_CO_3_/K_2_CO_3_	39/25/36	0.041	0.171	0.161	0.188

**Table 10:** Oxygen permeability rate mol·s^−1^·m^−2^) of ternary mixtures with the binary mixture Li_2_CO_3_/K_2_CO_3_ = 62/38 (%mol) as the basis for the temperature window of 500–650 °C.

Carbonate Mixture	Melt Composition (%mol)	500 °C	550 °C	600 °C	650 °C
		O_2_ Permeation Rate (mol·s^−1^·m^−2^)			
Li_2_CO_3_/K_2_CO_3_	62/38	−0.001	0.009	0.212	0.175
Li_2_CO_3_/Na_2_CO_3_/K_2_CO_3_	58.9/5/36.1	0.024	0.144	0.225	0.163
Li_2_CO_3_/Na_2_CO_3_/K_2_CO_3_	52.7/15/32.3	0.032	0.084	0.092	0.101
Li_2_CO_3_/Na_2_CO_3_/K_2_CO_3_	46.5/25/28.5	0.028	0.069	0.242	0.267

**Table 11:** O_2_ permeability rate (mol·s^−1^·m^−2^) of ternary eutectic mixtures and the same mole ratio of the three carbonates for the temperature window of 500–650 °C.

Carbonate Mixture	Melt Composition (%mol)	500 °C	550 °C	600 °C	650 °C
		O_2_ Permeation Rate (mol·s^−1^·m^−2^)			
Li_2_CO_3_/Na_2_CO_3_/K_2_CO_3_	43.5/31.5/25	0.014	0.029	0.138	0.224
Li_2_CO_3_/Na_2_CO_3_/K_2_CO_3_	33.3/33.3/33.3	0.018	0.035	0.133	0.161

## Data Availability

The original contributions presented in the study are included in the article, further inquiries can be directed to the corresponding author.

## Appendix A

Table A1 summarizes all the symbols and notations used in this paper.

**Table A1:** Symbols and notations used in this paper.

Definition	Symbol	Unit
Concentration	C	mol L^−1^
External surface	A	m^2^
Factor lambda	λ	ratio
Flux	J	cm^3^ min^−1^
Molar volume	V_m_	cm^3^ mol^−1^
Partial pressure	p	kPa
Permeation rate	r	mol s^−1^ m^−2^
